# Review and Analysis of Publication Trends over Three Decades in Three High Impact Medicine Journals

**DOI:** 10.1371/journal.pone.0170056

**Published:** 2017-01-20

**Authors:** Alexander Ivanov, Beata A. Kaczkowska, Saadat A. Khan, Jean Ho, Morteza Tavakol, Ashok Prasad, Geetha Bhumireddy, Allan F. Beall, Igor Klem, Parag Mehta, William M. Briggs, Terrence J. Sacchi, John F. Heitner

**Affiliations:** Department of Medicine, NewYork-Presbyterian Brooklyn Methodist Hospital, Brooklyn, New York, United States of America; Universidad de las Palmas de Gran Canaria, SPAIN

## Abstract

**Context:**

Over the past three decades, industry sponsored research expanded in the United States. Financial incentives can lead to potential conflicts of interest (COI) resulting in underreporting of negative study results.

**Objective:**

We hypothesized that over the three decades, there would be an increase in: a) reporting of conflict of interest and source of funding; b) percentage of randomized control trials c) number of patients per study and d) industry funding.

**Data sources and Study Selection:**

Original articles published in three calendar years (1988, 1998, and 2008) in The Lancet, New England Journal of Medicine and Journal of American Medical Association were collected.

**Data Extraction:**

Studies were reviewed and investigational design categorized as prospective and retrospective clinical trials. Prospective trials were categorized into randomized or non-randomized and single-center or multi-center trials. Retrospective trials were categorized as registries, meta-analyses and other studies, mostly comprising of case reports or series. Study outcomes were categorized as positive or negative depending on whether the pre-specified hypothesis was met. Financial disclosures were researched for financial relationships and profit status, and accordingly categorized as government, non-profit or industry sponsored. Studies were assessed for reporting COI.

**Results:**

1,671 original articles were included in this analysis. Total number of published studies decreased by 17% from 1988 to 2008. Over 20 year period, the proportion of prospective randomized trials increased from 22 to 46% (p < 0.0001); whereas the proportion of prospective non-randomized trials decreased from 59% to 27% (p < 0.001). There was an increase in the percentage of prospective randomized multi-center trials from 11% to 41% (p < 0.001). Conversely, there was a reduction in non-randomized single-center trials from 47% to 10% (p < 0.001). Proportion of government funded studies remained constant, whereas industry funded studies more than doubled (17% to 40%; p < 0.0001). The number of studies with negative results more than doubled (10% to 22%; p<0.0001). While lack of funding disclosure decreased from 35% to 7%, COI reporting increased from 2% to 84% (p < 0.0001).

**Conclusion:**

Improved reporting of COI, clarity in financial sponsorship, increased publication of negative results in the setting of larger and better designed clinical trials represents a positive step forward in the scientific publications, despite the higher percentage of industry funded studies.

## Introduction

Clinical research is vital to the evolution and practice of medicine. Fostered by support from both public and private sectors, there has been substantial growth in scientific innovations through clinical research. Contributions from industry have expanded considerably; however, the public sectors have not seen the same growth. Between 1994 and 2003 in the United States industry sponsorship of clinical trials increased from 4 to 14 billion dollars and it consistently accounts for the majority of funding for biomedical research (57%), with NIH as the next largest federal funder at 28% [1,2]. The remaining contribution is provided by state and local government (5%), non-NIH federal sources (5%), and private non-for-profit support (4%), which has not significantly changed since 1994 [1–3]. Over the years, industry funding towards clinical trials (phases 1–4) has increased from 33% to 52%, while federal proportions remained unchanged, from 43% to 45% for the same time period [1,3]. However, since 2003, the rate of increase of overall research spending has slowed overall. After a decade of doubling, the annual growth rate decreased from 7.8% between 1994 to 2003, to 3.4% between 2003 and 2008. [3]. As this increase in industry funded research develops, questions remain as to the effects on bias including study design, suppression of negative studies and favoring studies with positive outcomes [4–10].

Increased awareness of potential pitfalls has sounded a renewed commitment to the application of high ethical standards through increased oversight and awareness. Establishment of hospital institutional review boards, scrutiny over disclosure of conflict of interest (COI), registration of clinical trials and public access to study information have been implemented to monitor for potential COI)[7]. Thus, we sought to assess trends in clinical study design, sponsorship sources, study outcomes, and reporting of COI within the past three decades, as reported within three prominent peer-reviewed journals.

## Methods

We chose one calendar year from each of the past three decades as a sample year for analysis. All original articles published during the calendar year of 1988, 1998 and 2008 from three leading peer-reviewed journals: The New England Journal of Medicine (NEJM), Lancet and Journal of American Medical Association (JAMA) were included. We excluded editorials and letters to editors. These journals were chosen because they represent the most widely circulated English-language journals publishing original research to general clinical practitioners. Each article was reviewed and categorized according to study design, source of funding, disclosure of author affiliations and study outcomes. If there was any question or doubt on category assignment, the question was brought to the entire group and a decision was rendered by consensus. This occurred in less than 2% of the total articles reviewed. The definitions for these parameters were specified prior to investigator analysis of the articles. There was no external funding to support this work.

### Definitions

All original articles were chosen and assessed by three separate reviewers, each assigned to review one peer-reviewed journal. The studies were categorized according to specific predetermined definitions that were reviewed by the team before the start of the study. The article was first categorized according to its temporal characteristics: prospective or retrospective. A *prospective study* is one that is conducted simultaneously with the events under study and the hypothesis is conceived prior to patient enrollment. The patients are followed forward in time and the data is gathered at baseline and as the events happen. A *retrospective study* is one that is carried out after the events under study have occurred, with data collection and assessment occurring prior to study hypothesis. Prospective studies were further categorized as randomized or non-randomized. A randomized study or randomized clinical trial (RCT) incorporates chance assignment of participants to different groups, one of which is a control or comparator group. Non-RCT studies included those that used data from non-randomized prospective cohorts (observational survey) as well as longitudinal studies that were not controlled. Prospective studies were further sub-categorized with relation to the number of participating centers involved in conducting the studies. When more than one institution was involved in enrolling and recruiting patients, the study was charted as a multicenter study. Studies conducted entirely at one center were recorded as single center studies. Retrospective studies included the following: (1) case-control studies, where subjects are chosen because of a particular outcome and followed backward in time from outcome to possible cause which is derived by comparing diseased patients (cases) with non-diseased patients (controls); (2) registry studies (3) and meta-analyses.

Source of funding was assessed by review of methods and end-notes of the published study, when reported. Identification of the funding source was followed by an extensive on-line search for the financial relationships and profit status of the organization. Industry sponsors were companies or associations that reported a for-profit commitment in their mission statement. Non-profit sponsorship was used to denote non-profit, charitable institutions that explicitly stated a non-profit relationship in their description of their organization. Government sponsors would have reported their relationship in association with the country in which the study was conducted. Reporting of COI was classified as positive if any conflicts were reported, negative if no conflicts were specifically reported in the article, and none if there was no mention of COI. When more than one source of sponsorship was reported, the financial relationship was counted as multiple, and single when only one source was reported. Study outcomes were reported as positive if they met the pre-specified null hypothesis was rejected, negative if they failed to do so.

### Hypothesis

We hypothesized that over the three decades, there would be an increase in: a) reporting of conflict of interest and source of funding; b) randomized control trials and number of patients per study and c) industry funding.

### Statistical analysis

We used R version 3.1.2 (R Foundation for Statistical Computing, Vienna, Austria). Difference <0.05 was considered significant. We calculated summary statistics for the various study types by year were. Since we observe non-Gaussian distribution in all analyzed variables we used Wilcoxon test for the analysis of differences between category proportions, as well as for differences between median patient numbers. Inter-observer reliability was assessed using a total of twenty articles from the specified journals and categorized by all three reviewers and statistically characterized by Fleiss’s kappa.

## Results

**Fig 1:**
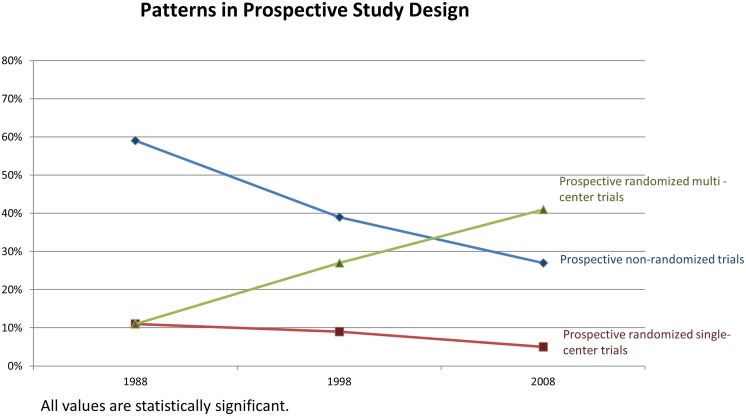
Patterns in trial design.

A total of 1,671 original articles were reviewed from 1988, 1998, and 2008. Within the pre-specified calendar years, the proportion of prospective and retrospective studies did not change considerably, 483 (80%), 422 (74%), 370 (74%), respectively, for prospective studies and 118 (20%), 146 (26%), 132 (26%) respectively, for retrospective studies (Table 1). However, there was an absolute decrease by 17% of all studies (from 601 to 502) published between 1988 and 2008. There was a significant, nearly double, shift toward publication of prospective randomized trials, increasing from 131 (22%) in 1988 to 232 (46%) in 2008 (p < 0.0001). An increase in the proportion of prospective randomized multicenter trials was noted, comprising 11% of studies published in 1988, compared to 41% in 2008, (p < 0.0001) (Table 1). Conversely, over the last three decades, there was a reduction from 11% to 5% in randomized single center studies and a reduction from 59% to 27% in prospective non-randomized studies. In particular, there was an absolute reduction by 37% of prospective non-randomized single center studies, from 47% in 1988 to 10% in 2008 (p < 0.0001) (Fig 1). Amongst retrospective studies, a higher proportion of studies were registries and meta-analyses, with an increase total number of registries from 9% in 1988 to 14% in 2008 (p = 0.002) (Table 1). In addition, there was greater than tenfold increase, though not statistically significant, in the median number of patients per study, 103 to 1380 from 1988 to 2008, (p = 0.2)).

**Table 1:** Characteristics of the included studies.

	1988 (%)	1998 (%)	2008 (%)	P Value
STUDY DESIGN	N = 601	N = 568	N = 502	
*PROSPECTIVE STUDIES*	483 (80%)	422 (74%)	370 (74%)	0.013
Prospective randomized	131 (22%)	203 (36%)	232 (46%)	< 0.0001
Prospective non-randomized	352 (59%)	219 (39%)	138 (27%)	< 0.0001
Prospective randomized single-center	67 (11%)	49 (9%)	25 (5%)	0.001
Prospective randomized multi-center	64 (11%)	154 (27%)	207 (41%)	< 0.0001
Prospective non-randomized single-center	282 (47%)	99 (17%)	52 (10%)	< 0.0001
Prospective non-randomized multi-center	70 (12%)	120 (21%)	86 (17%)	< 0.0001
*RETROSPECTIVE STUDIES*	118 (20%)	146 (26%)	132 (26%)	0.013
Registry	52 (9%)	51 (9%)	73 (14%)	0.002
Meta-analysis	13 (2%)	8 (2%)	18 (4%)	0.059
Other *	53 (9%)	87 (15%)	41 (8%)	0.0001
Study Size (median number of patients)	103	595	1380	0.20
FUNDING				
Reported funding	388 (65%)	471 (83%)	469 (93%)	
Government	288 (74%)	339 (72%)	326 (70%)	0.31
Industry	65 (17%)	130 (28%)	186 (40%)	< 0.0001
Non-profit	167 (43%)	177 (38%)	188 (40%)	0.27
No funding / None reported	213 (35%)	97 (17%)	33 (7%)	< 0.0001
Single funding	234 (39%)	199 (35%)	172 (34%)	0.22
Multiple funding	154 (26%)	272 (48%)	297 (59%)	< 0.0001
Disclosure of positive COI	10 (2%)	34 (6%)	280 (56%)	< 0.0001
Disclosure of negative COI	0 (0%)	4 (1%)	141 (28%)	<0.0001
No disclosure provided	591 (98%)	530 (93%)	81 (16%)	<0.0001
OUTCOMES				
Positive	543 (90%)	477 (84%)	394 (78%)	< 0.0001
Negative	58 (10%)	91 (16%)	108 (22%)	< 0.0001

* case control studies, cross-sectional studies, case reports

**Fig 2:**
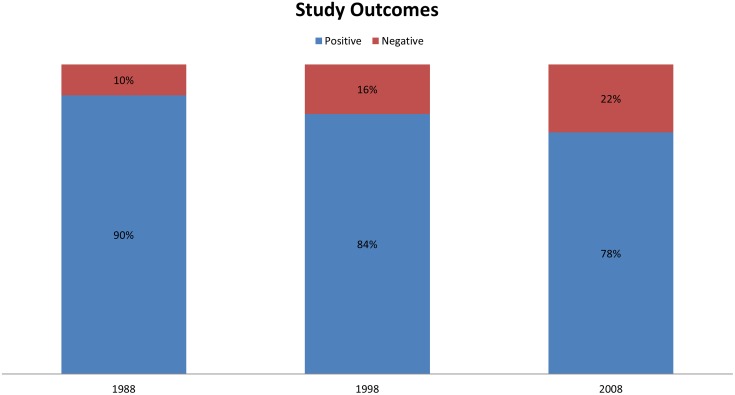
Study outcomes amongst all published articles within calendar years.

The proportion of reported government and non-profit funding did not change significantly across the calendar years, whereas a significant increase in industry funding was noted, from 17% in 1988 to 40% in 2008, (p < 0.0001) (Fig 2). Statistically greater utilization of multiple funding sources were also reported in the later years, from 26% to 59% (P < 0.001) (Table 1). In 1988, 35% of the studies either failed to comment on funding source or reported no funding, as opposed to only 7% of the studies in 2008. The shifts in reporting of funding source were accompanied by significant increases in reported COI. Only 2% of the authors had reported on either the presence or absence of COI in 1988, compared to 84% of the authors in 2008 (p < 0.0001). Studies with positive outcomes did not increase in parallel with higher industry involvement. In fact, the proportion of studies reporting negative outcomes increased steadily from 1988 to 2008 (10% vs. 22%, respectively, p < 0.0001) (Table 1).

Sub-analysis of the relationship between prospective multicenter RCTs and industry sponsorship showed an increase in interaction between 1988 and 2008. There was a significant reduction in reporting of positive outcomes from 83% to 69%, along with a concomitant increase in industry funding from 31% to 64% as well as increase in reporting of COI from 3% to 77% (Table 2).

**Table 2:** Important features of the multicenter randomized clinical trials.

	1988 (%)	1998 (%)	2008 (%)	P Value
PROSPECTIVE RANDOMIZED MULTI-CENTER TRIALS	N = 64	N = 154	N = 207	
Industry sponsorship	20 (31%)	78 (51%)	133 (64%)	< 0.0001
Positive outcomes	53 (83%)	124 (81%)	143 (69%)	0.015
Conflict of interest	2 (3%)	26 (17%)	159 (77%)	< 0.0001
Study Size (Median number)	280	570	940	p = 0.12

Inter-observer reliability was assessed using a total of twenty articles from the specified journals and categorized by all three reviewers. A mean kappa value was calculated to be 0.98 (p-value < 0.00001), indicating absence of significant interobserver variability.

## Discussion

The current analysis of publication trends examines changes in research methodology and COI within the modern era of growing industry involvement amongst leading medical journals. We have shown a paradigm shift in publication from small single center studies to large multicenter RCTs. The trend toward higher level of research design has been met with improved disclosure of COI and publication of studies with negative outcomes. This pattern demonstrates a positive step towards clarity in disclosure and conduct amongst clinical studies.

The relationship between medical research and for-profit organizations has expanded considerably over the past thirty years. The Bayh-Dole Act of 1980 opened the door to an era of cooperation between industry and biomedical research [11]. Since then, industry sponsorship has grown exponentially, eventually overtaking the federal government in the early 90’s as the largest contributor of medical research in the United States [12,13]. In 2011, investment in research and development by members of the Pharmaceutical Research and Manufacturers of America was estimated at $49.5 billion which is nearly double the $26.4 billion in 2000, and exponentially more than the $2 billion spent in 1990 [12,13]. Government funding of biomedical research has not expanded at the same pace. Between 1994 and 2004, the relative proportion of government and private contributions did not change, whereas industry sponsorship of clinical trials more than tripled [1,2].

There is also evidence that industry has sponsored the most prominent studies and summary submissions, as well as, helps support many of the thought leaders in academia [8,14]. In analysis of the most frequently cited RCTs from 1994 to 2003, over half (56%) were funded by industry alone [1]. In surveys of participants of 17 practice guideline documents from the American Heart Association and American College of Cardiology published from 2004 to 2008, 56% of the authors listed in the guidelines development process had disclosed potential COI with industry [15].

The expanding role of industry in biomedical research can have numerous ramifications. Multiple sources of bias can be encountered when financial gain is dependent on positive clinical trial outcome. This evidenced by authors with financial COI implicated in being more likely in arriving at positive study conclusions. In their landmark study in 1998, Stelfox et. al, showed that 96% of the supportive authors compared to 37% of the critical authors for safety of calcium channel blockers had some form of financial relationship with the drug manufacturer [16]. As depicted in a systemic review, numerous other studies since then have reported similar findings of systematic bias favoring products which are made by the company funding the research [17]. Financial conflicts are more likely to be reported if they are related to the topic of presentation, involve large financial sums, or paid directly to individuals. In a study of payment made to physicians by manufacturers of orthopedic prostheses, the rate of non-disclosure was 21% for directly related payments and 50% for indirectly related payments (1[13]. Financial COI have also been implicated in bias study design and collection of data, especially with the involvement of for-profit contract research organizations that are hired by the industry to conduct clinical trials (4)[9]. Other reported sources of bias include suppression of negative results, publication delay, and restrictions on investigator behavior (8).

Within the academic community, the increasing awareness of the financial stakes for industry has brought with it a welcomed scrutiny over COI. The renewed focus on maintaining ethical integrity has introduced provisions to address industry related COI. Medical journals were the first organizations to develop policies to address COI. This was initiated with the New England Journal of Medicine in 1984 and the Journal of the American Medical Association in 1985. Starting in the early 1990s a number of measures were taken to disclose objectivity in research. It is worth noting, that number of studies that reported on COI rose steadily over the decades in each journal, with the largest increase between 1998 and 2008. However, in NEJM these major changes are noted a little earlier, between 1988 and 1998, suspect this is probably a reflection of the earlier adaptation of COI guidelines in 1984 by this journal. This will be added to the discussion section In 1995, investigators applying for funding from public health services were required to disclose any significant COI that could potentially affect their research. This was followed by FDA requirements for investigators to disclose a pre-specified significant amount of financial interest in their application for approval of a new drug or device [18]. Starting in early 2000’s, development and implementation of publication guidelines have played an increasingly prominent part in the biomedical literature to improve reporting quality and contributes greatly to clarity and transparency in the reporting of research (16)[14]. Guidelines have been developed for reporting meta-analysis and systematic reviews of randomized controlled trials (QUOROM) in 1999, meta-analysis and systematic review of observational studies (MOOSE) in 2000, randomized controlled trials (CONSORT) in 2001, epidemiological observational studies (STROBE) in 2002, and for other designs and content areas including quality improvement measures such as the standards for quality improvement reporting excellence (SQUIRE) in 2008 with most recent update just published in 2016.[19] [20]. Establishment of a clinical trial databank through the Modernization Act of 1997 empowered the NIH to establish, maintain, and operate a data bank of information on clinical trials for drugs to treat serious or life-threatening diseases and conditions (14)[18]. The Clinical Trials Data Bank provides a central resource of updated information on clinical trials to members of the public, and to health care providers and researchers. The American Medical Association and the American College of Cardiology also promote disclosure and encourage individual centers to develop specific guidelines for their clinical staff. This was followed by the publication of the first PRISMA statement in 2009 summarizing evidence-based minimum set of items for reporting in systematic reviews and meta-analyses and PRISMA-P statement in 2015, setting similar recommendations for network meta-analysis[21,22].

Early analysis of the systematic reviews and meta-analysis evaluating 175 papers by Jørgensen et al. published in 2006, found that industry sponsored reviews to be less transparent, had few reservations about methodological limitations of the analyzed studies, and had more favorable conclusions compared to studies independently performed[10]. In 2012 Lundh et al. published a Cochrane Review suggesting that industry studies are 24% more likely to find favorable outcomes, were 87% less likely to report harm and, overall, were 31% more likely to present a favorable conclusion compared with studies having other sources of sponsorship. They also found that despite no difference in a standard risk of bias, Industry-sponsored trials are more often favorable to the sponsor’s products compared with non–industry-sponsored trials because of trial specific biases that cannot be explained by standard “risk of bias”. Authors of Cochrane review called to update current tools to asses bias and to consider industry sponsorship as a bias [4]. Another systematic review investigating reviews of the effects of artificially sweetened beverages on weight found that sponsorship and authors’ financial COI introduced bias influencing the outcomes of reviews that could not be explained by other sources of bias. Although our study found an increase in negative studies published over time, the large majority of studies published (78% in 2008) were positive studies[6].

We have also found a relative increase in the publication of studies with negative outcomes. This might be attributed to higher level of evidence studies in the form of RCTs, which also have increased over time. It is plausible that the increase in industry funding allows for the larger number of RCT’s that were performed over time, however, this is an association and we cannot draw conclusions as to causality.”However, increased public awareness, disclosure requirements, and the inquiry over financial relationships to study outcomes should not be discounted as driving forces for changing research trends. There is still substantial growth and oversight needed in order to continue the current trend toward clarity in research conduct.

The current study does have some potential limitations. We sampled three high impact journals and our findings therefore cannot be generalized to all medical journals. The articles from each journal were interpreted by three separate reviewers and may suffer from inter-observer variability. In order to limit this, the definitions for categorizing the studies were made clear to the investigators prior to initiating the study. In addition, the inter-observer variability on a subset of studies indicated an excellent agreement. The affiliation of all the authors and the magnitude of involvement with their funding source were not possible given the large number of studies involved. In addition, although there are positive trends noted in this manuscript, other potential biases from industry sponsorship of medical research such as the “framing” of the results, could not be assessed. Important changes in policies and practices of reporting conflict of interest should be noted: NEJM adopted a requirement to report COI in 1984, JAMA in 1985, and we were unable to obtain the date of adoption for the LANCET. More recently, the Sunshine act first proposed in 2007 and later implemented in 2010 as a part of Affordable care act, the US government required industry to report any financial relationships with physicians participating in Medicaid/Medicare/State Children's Health Insurance Program, and to report these relationships on-line in a free, accessible database.

## Conclusion

Improved reporting of COI, clarity in financial sponsorship, increased likelihood of publication of negative results in the setting of larger, and better designed clinical trials represents a positive step forward in the scientific publications despite higher reliance on industry funded studies.

## Supporting Information

S1 Fig Systematic review checklist. PRISMA 2009 checklist.
(DOC)

S1 Table Studies published in NEJM. Raw data NEJM.
(XLS)

S2 Table Studies published in JAMA Raw data JAMA. (XLS)

S3 Table Studies published in LANCET. Raw data LANCET.
(XLS)

## References

[1] Moses H 3rd, Dorsey ER, Matheson DH, Thier SO (2005) Financial anatomy of biomedical research. JAMA 294: 1333–1342. 10.1001/jama.294.11.1333 16174691

[2] Moses H 3rd, Matheson DH, Cairns-Smith S, George BP, Palisch C, Dorsey ER (2015) The anatomy of medical research: US and international comparisons. JAMA 313: 174–189. 10.1001/jama.2014.15939 25585329

[3] Dorsey ER, de Roulet J, Thompson JP, Reminick JI, Thai A, White-Stellato Z, et al (2010) Funding of US biomedical research, 2003–2008. JAMA 303: 137–143. 10.1001/jama.2009.1987 20068207 PMC3118092

[4] Lundh A, Sismondo S, Lexchin J, Busuioc OA, Bero L (2012) Industry sponsorship and research outcome. Cochrane Database Syst Rev 12: MR000033 10.1002/14651858.MR000033.pub2 23235689

[5] Norris SL, Holmer HK, Ogden LA, Burda BU, Fu R (2012) Characteristics of physicians receiving large payments from pharmaceutical companies and the accuracy of their disclosures in publications: an observational study. BMC Med Ethics 13: 24 10.1186/1472-6939-13-24 23013260 PMC3507829

[6] Mandrioli D, Kearns CE, Bero LA (2016) Relationship between Research Outcomes and Risk of Bias, Study Sponsorship, and Author Financial Conflicts of Interest in Reviews of the Effects of Artificially Sweetened Beverages on Weight Outcomes: A Systematic Review of Reviews. PLoS One 11: e0162198 10.1371/journal.pone.0162198 27606602 PMC5015869

[7] Morin K, Rakatansky H, Riddick FA Jr., Morse LJ, O'Bannon JM 3rd, Goldrich MS, et al (2002) Managing conflicts of interest in the conduct of clinical trials. JAMA 287: 78–84. 11754712 10.1001/jama.287.1.78

[8] Als-Nielsen B, Chen W, Gluud C, Kjaergard LL (2003) Association of funding and conclusions in randomized drug trials: a reflection of treatment effect or adverse events? JAMA 290: 921–928. 10.1001/jama.290.7.921 12928469

[9] Bekelman JE, Li Y, Gross CP (2003) Scope and impact of financial conflicts of interest in biomedical research: a systematic review. JAMA 289: 454–465. 12533125 10.1001/jama.289.4.454

[10] Jorgensen AW, Hilden J, Gotzsche PC (2006) Cochrane reviews compared with industry supported meta-analyses and other meta-analyses of the same drugs: systematic review. BMJ 333: 782 10.1136/bmj.38973.444699.0B 17028106 PMC1602036

[11] (1980) Bayh-Dole Act. Pub L No 35 USSC: 96–517.

[12] Okike K, Kocher MS, Mehlman CT, Bhandari M (2008) Industry-sponsored research. Injury 39: 666–680. 10.1016/j.injury.2008.02.013 18508054

[13] Okike K, Kocher MS, Wei EX, Mehlman CT, Bhandari M (2009) Accuracy of conflict-of-interest disclosures reported by physicians. N Engl J Med 361: 1466–1474. 10.1056/NEJMsa0807160 19812403

[14] Ogrinc G, Mooney SE, Estrada C, Foster T, Goldmann D, Hall LW, et al (2008) The SQUIRE (Standards for QUality Improvement Reporting Excellence) guidelines for quality improvement reporting: explanation and elaboration. Qual Saf Health Care 17 Suppl 1: i13–32. 18836062 10.1136/qshc.2008.029058 PMC2602740

[15] Neuman J, Korenstein D, Ross JS, Keyhani S (2011) Prevalence of financial conflicts of interest among panel members producing clinical practice guidelines in Canada and United States: cross sectional study. BMJ 343: d5621 10.1136/bmj.d5621 21990257 PMC3191201

[16] Stelfox HT, Chua G, O'Rourke K, Detsky AS (1998) Conflict of interest in the debate over calcium-channel antagonists. N Engl J Med 338: 101–106. 10.1056/NEJM199801083380206 9420342

[17] Lexchin J, Bero LA, Djulbegovic B, Clark O (2003) Pharmaceutical industry sponsorship and research outcome and quality: systematic review. BMJ 326: 1167–1170. 10.1136/bmj.326.7400.1167 12775614 PMC156458

[18] (1998) Financial disclosure by clinical investigator. Fed Regist 63. 10177332

[19] Goodman D, Ogrinc G, Davies L, Baker GR, Barnsteiner J, Foster TC, et al (2016) Explanation and elaboration of the SQUIRE (Standards for Quality Improvement Reporting Excellence) Guidelines, V.2.0: examples of SQUIRE elements in the healthcare improvement literature. BMJ Qual Saf. 10.1136/bmjqs-2015-004480 PMC5256235 27076505

[20] Mendelson TB, Meltzer M, Campbell EG, Caplan AL, Kirkpatrick JN (2011) Conflicts of interest in cardiovascular clinical practice guidelines. Arch Intern Med 171: 577–584. 10.1001/archinternmed.2011.96 21444849

[21] Hutton B, Salanti G, Caldwell DM, Chaimani A, Schmid CH, Cameron C, et al (2015) The PRISMA extension statement for reporting of systematic reviews incorporating network meta-analyses of health care interventions: checklist and explanations. Ann Intern Med 162: 777–784. 10.7326/M14-2385 26030634

[22] Shamseer L, Moher D, Clarke M, Ghersi D, Liberati A, Petticrew M, et al (2015) Preferred reporting items for systematic review and meta-analysis protocols (PRISMA-P) 2015: elaboration and explanation. BMJ 349: g7647 10.1136/bmj.g7647 25555855

